# Vitamin K Status and Skeletal Fragility: Differential Associations of Osteocalcin Carboxylation States with Bone Strength, Geometry and Microarchitecture

**DOI:** 10.3390/nu18162663

**Published:** 2026-08-14

**Authors:** Deepti K. Sharma, Chloe Furst, Rebecca Bahnisch, Christopher Schultz, Manuela Rogers, Tim Cheok, Lucian B. Solomon, Stuart A. Callary, Boopalan Ramasamy

**Affiliations:** 1 Department of Orthopaedics and Trauma, Royal Adelaide Hospital, Adelaide, SA 5000, Australia; bogdan.solomon@sa.gov.au (L.B.S.); stuart.callary@sa.gov.au (S.A.C.); boopalan.ramasamy@adelaide.edu.au (B.R.); 2 Adelaide University Rural Health School, College of Health, Adelaide University, Adelaide, SA 5000, Australia; 3 Department of Geriatrics, Royal Adelaide Hospital, Adelaide, SA 5000, Australia; chloe.furst@sa.gov.au; 4 Chemical Pathology, South Australia Pathology, Adelaide, SA 5000, Australia; rebecca.bahnisch@sa.gov.au; 5 Department of Nuclear Medicine, Royal Adelaide Hospital, Adelaide, SA 5000, Australia; cg_schultz@outlook.com.au; 6 Discipline of Orthopaedics and Trauma, School of Medicine, College of Health, Adelaide University, Adelaide, SA 5005, Australia; manuela.rogers@student.adelaide.edu.au; 7 College of Medicine and Public Health, Flinders University, Adelaide, SA 5001, Australia; tim.cheok@flinders.edu.au

**Keywords:** hip fracture, vitamin K, osteocalcin carboxylation, skeletal fragility, bone microarchitecture

## Abstract

**Background:** The contribution of specific nutritional biomarkers to skeletal fragility remains poorly understood, in part because most studies include participants with metabolic conditions that obscure nutrient-specific effects. We aimed to examine incremental contribution of six pathway-specific nutritional biomarkers. **Methods**: In a prospective cross-sectional case–control design, 108 patients undergoing arthroplasty were enrolled (hip-fracture, *n* = 63; non-fracture, *n* = 45), and bone biopsies and blood specimens were collected intraoperatively. Circulating biomarkers reflecting vitamin K, one-carbon metabolism, antioxidant nutrients (vitamins E and C), protein and zinc status were measured. Bone outcomes included remodelling markers, trabecular microarchitecture and bone strength. Hierarchical regression was used to quantify the incremental contribution of nutritional biomarkers beyond clinical covariates with a priori outcome selection. **Results:** Bone remodelling markers did not differ between groups after covariate adjustment, indicating that fractures in this cohort were characterised by deficits in bone strength and quality rather than elevated systemic remodelling activity. Among six nutritional pathways tested, only vitamin K-dependent biomarkers showed consistent independent associations with bone outcomes, suggesting a degree of pathway specificity. Critically, carboxylated (cOC), undercarboxylated (ucOC) and fully uncarboxylated (unOC) osteocalcin fractions showed differential associations reflecting their distinct biological roles: cOC was independently associated with greater bone strength (section modulus, femoral neck width and cortical shaft thickness; *p* < 0.05) and lower trabecular separation (Tb.Sp); ucOC was associated with higher Tb.Sp (*p* = 0.004); and unOC was positively associated with cortical bone instability (buckling ratio, *p* = 0.003) and explained 17% of the variance in the bone resorption marker (*p* < 0.001), consistent with a shift towards bone loss. Vitamin K2-7 was negatively associated with hip axis length (*p* = 0.021). **Conclusions**: These findings identify vitamin K-dependent carboxylation as a mechanistically specific and modifiable factor associated with skeletal fragility beyond bone mineral density, with distinct skeletal consequences across osteocalcin carboxylation states.

## 1. Introduction

Osteoporotic fractures are a major public health concern, causing substantial morbidity, mortality and healthcare burden worldwide [1]. These fractures are the result of multiple interacting factors, including age; female sex; genetics; bone mineral density (BMD); fall risk [2]; or conditions that affect bone remodelling such as diabetes [2,3,4], chronic kidney disease (CKD) [2,5,6], primary or secondary hyperparathyroidism [2,7], chronic glucocorticoid use [2] and cancer [2]. However, fractures may also occur in individuals without identifiable metabolic risk factors, suggesting that additional modifiable contributors, including nutritional pathways, warrant investigation.

Nutrition is a key modifiable factor that is known to play a role in bone remodelling and structural integrity [8,9]. While epidemiological studies have reported associations between dietary patterns and bone outcomes including BMD and microarchitecture [10,11,12,13,14,15,16,17], findings have been inconsistent [18], and such approaches fail to clarify the specific mechanisms by which nutrients influence skeletal outcomes [8,9,19]. A recent bibliometric analysis demonstrates that the literature has predominantly focused on dietary intake measures rather than biochemical nutrient biomarkers, metabolic pathways or dynamic indices of skeletal metabolism [20]. To overcome these issues, several expert groups have recommended the use of biomarkers that reflect a biological response to changes in nutrient levels or dietary patterns [19,21]. Nutritional biomarkers are objectively measured functional or clinical indices of nutrient intake or status that can provide important information on the normal physiological response and pathogenic processes in the setting of nutritional inadequacies and are responsive to intervention [22].

To our knowledge, no prior study has comprehensively examined the contributions of pathway-specific nutritional biomarkers to functional skeletal outcomes in adults without overt metabolic or endocrine diseases. Existing vitamin K-bone research has primarily used total or circulating vitamin K concentrations, without distinguishing the functional consequences of different osteocalcin carboxylation states on discrete aspects of bone quality. Our recent work demonstrated that carboxylated osteocalcin independently predicts femoral bone strength indices in patients without metabolic disorders [23]; however, that study was restricted to the non-fracture group and did not examine the differential contributions of undercarboxylated or fully uncarboxylated fractions, nor their associations with bone microarchitecture or turnover. Accordingly, the primary aim was to quantify the independent and incremental associations of all three-vitamin K-dependent osteocalcin fractions, namely, carboxylated (cOC), undercarboxylated (ucOC) and fully uncarboxylated (unOC), with bone microarchitecture, strength and turnover across both fracture and non-fracture patients without clinically apparent metabolic disorders. Based on our previous findings demonstrating independent associations between cOC and femoral bone strength in non-fracture patients [23], we hypothesised that vitamin K-dependent osteocalcin carboxylation would independently predict bone quality outcomes in a combined fracture and non-fracture cohort. We further hypothesised that examining all three carboxylation states (cOC, ucOC and unOC) would reveal differential associations with distinct aspects of bone quality. The secondary aim was to examine the specificity of these associations within a comprehensive framework of six nutritional biomarker pathways, including one-carbon metabolism, antioxidant nutrients, protein, zinc and vitamin D–calcium homeostasis. Given our previous findings that elevated homocysteine is associated with bone quality measures beyond BMD, including bone turnover and collagen crosslinking [24], and that higher 25-hydroxyvitamin D is associated with improved bone formation and micro-structural measures in elderly hip fracture patients [25], we further hypothesised that one-carbon metabolism and vitamin D–calcium homeostasis would also contribute independently to bone quality in this cohort.

## 2. Materials and Methods

### 2.1. Subjects

This prospective cross-sectional case–control study included 108 consecutive patients who presented to the Royal Adelaide Hospital between April 2022 and February 2024 for treatment of (1) fragility hip fracture (*n* = 63, fracture group) and (2) hip osteoarthritis (*n* = 45, non-fracture group). Patients were excluded if they had CKD, diabetes, primary or secondary hyperparathyroidism, inflammatory arthritis, inflammatory bowel disease or cancer; if they had elevated alkaline phosphatase (ALP) >110 U/L; or if they were taking medications known to affect bone metabolism, including glucocorticoids, antiresorptive agents or warfarin (Table 1). Exclusion of hyperparathyroidism was based on clinical diagnosis documented in medical records, as biochemical values were only available following enrolment and intraoperative blood collection. All biochemical values reported in this study, including ALP, reflect intraoperative measurements collected at the time of surgery for consistency across all biomarkers. Published evidence demonstrates that ALP remains normal on admission in the majority of hip fracture patients, only rising 7–9 days post-fracture [26], confirming that intraoperative values reflect the pre-fracture metabolic state. Patients with BMI > 38 kg/m^2^ were excluded due to the limitations of DXA equipment at our institution to reliably measure body composition above this threshold. This study was conducted in accordance with the Declaration of Helsinki and approved by the Central Adelaide Local Health Network Human Research Ethics Committee (CALHN-HREC) (approval code: 15811; approval date: 16 February 2022). All participants provided written informed consent before being recruited. Intertrochanteric bone cores and fasting blood specimens were collected intraoperatively. The bone cores were fixed in phosphate-buffered formaldehyde for 7 days, followed by submersion in 70% ethanol until further analysis. Blood was centrifuged at 3000 rpm for 15 min at 4 °C within 2 h of collection and serum was stored at −80 °C until analysis.

### 2.2. Nutritional Biomarkers

The selection of nutritional biomarkers in this study was guided by current evidence on the micronutrients and metabolic pathways most relevant to bone turnover, microarchitecture and strength. Accordingly, biomarkers were grouped into six physiologically relevant pathways: (1) vitamin K-dependent carboxylation, which included phylloquinone or vitamin K1; menaquinone-7 or vitamin K2-7; and the functional biomarkers of vitamin K status, i.e., carboxylated osteocalcin (cOC), uncarboxylated (unOC) and undercarboxylated osteocalcin (ucOC) [23,27]; (2) one-carbon metabolism (vitamin B6, B12 (and active B12, when required), folate and homocysteine), which supports DNA synthesis and methylation and collagen cross-linking [24]; (3) vitamin D–calcium homeostasis, including total calcium and 25-hydroxyvitamin D [25]; (4) antioxidant micronutrients (vitamins C and E), which influence collagen synthesis and oxidative stress within bone [28]; (5) protein status (measured as albumin levels) [29]; and (6) zinc levels [30]. Vitamin D sufficiency was defined as serum 25-hydroxyvitamin D ≥ 60 nmol/L, consistent with our institutional (SA Pathology) reference ranges. This threshold was used for descriptive reporting only, while serum 25-hydroxyvitamin D concentrations were analysed as a continuous variable in all regression models. Sensitivity analyses using the internationally recommended threshold of 50 nmol/L did not materially alter regression model results. All biomarkers were measured by NATA accredited laboratory, SA Pathology. ALP, magnesium and homocysteine were measured using routine colourimetric assays, while total calcium was measured using an NM-BAPTA assay; all analyses were performed on a cobas c 702 analysers (Roche Diagnostics GmbH, Mannheim, Germany). Albumin was measured using a bromocresol purple (BCP) assay on a cobas c 702 analysers (Roche Diagnostics GmbH). Vitamin B12 (B12), active B12 (holotranscobalamin), folate and parathyroid hormone (PTH) were measured using electrochemiluminescence immunoassays (ECLIA) on a cobas e 802 analysers (Roche Diagnostics GmbH). 1,25-dihydroxyvitamin D was measured using ECLIA assay performed on a Diasorin LIAISON XLAS analyser (DiaSorin S.p.A., Saluggia, Italy). Vitamins 25-hydroxyvitamin D, B1, B2 and B6 were measured by liquid chromatography–tandem mass spectrometry (LC-MS/MS), while vitamins C and E were measured by high-performance liquid chromatography (HPLC). Zinc was measured using an inductively coupled plasma mass spectrometry (ICP-MS) method. Vitamin K1 and menaquinone-7 (vitamin K2-7) were measured using a previously described HPLC method [23]. Carboxylated osteocalcin (cOC) and uncarboxylated osteocalcin (unOC) were measured by ELISA (Takara Bio USA, Inc., San Jose, CA, USA) [23]. The American Society of Anaesthesiologists (ASA) grades were retrieved from the clinical records.

### 2.3. Bone Turnover Markers

Nutritional deficiencies are known to influence bone turnover rate [8]. Markers recommended by the International Osteoporosis Foundation and International Federation of Clinical Chemistry and Laboratory Medicine (IOF-IFCC) were therefore measured [31]. Procollagen type I *N*-propeptide (PINP) is cleaved from type I procollagen during collagen synthesis and reflects osteoblastic collagen matrix production [31]. Bone-specific alkaline phosphatase (bone ALP) is an osteoblast enzyme involved in matrix mineralisation of newly formed osteoid and is a specific marker of osteoblast activity [31,32]. *C*-terminal telopeptide (CTX-1) is generated during osteoclastic degradation of type 1 collagen and reflects bone resorption [31]. Osteocalcin (OC) is the most abundant non-collagenous bone protein, synthesised by mature osteoblasts, and serum total OC is a marker of bone turnover [33]. PINP and CTX-1 were measured using ECLIA assays on a cobas e 802 analysers (Roche Diagnostics GmbH), while bone specific ALP and OC were measured using assays on a LIAISON XLAS analyser (DiaSorin S.p.A).

### 2.4. Trabecular Bone Microarchitecture Analysis Using microCT

Bone specimens were scanned using the SkyScan 1276 (Bruker Micro-CT, Kontich, Belgium) imaging system at a voxel size of 10 microns with a 0.5 mm aluminium filter. Region of interest selection, adaptive local thresholding, volume of interest wrapping, debris removal and morphometric analysis were performed using a validated, reproducible automated workflow described in detail elsewhere [34]. Trabecular bone microarchitecture parameters included bone volume to tissue volume (BV/TV), trabecular thickness (Tb.Th), trabecular number (Tb.N), trabecular pattern factor (Tb.Pf), trabecular separation (Tb.Sp), fractal dimension (FD), connectivity density (Conn.D) and Un-Plate Index.

### 2.5. Bone Geometry and Bone Strength Using Dual Energy X-Ray Absorptiometry

All patients underwent dual-energy X-ray absorptiometry (DXA, GE-Lunar Prodigy, Madison, WI, USA) to measure whole body, femur and spine BMD. DXA scans were performed preoperatively in the non-fracture group and on postoperative day 3 (or once clinically stable) on the contralateral side in the fracture group. Advanced Hip Analysis (AHA) software integrated into encore 18.5 was used to derive geometric and structural parameters including hip axis length (HAL); cortical thickness (CT) at the femoral neck, calcar and shaft; strength index; section modulus; cross-sectional moment of inertia (CSMI); cross-sectional area (CSA); buckling ratio; and femoral neck width, defined as double the distance from the center of femoral neck mass to the superior neck margin positioned at the location of the line from the femoral head center to the section of minimum CSMI [35]. Scans were analysed by one operator (CS). Physical activity was assessed using a validated self-reported Community Health Activities Model Program for Seniors (CHAMPS) questionnaire [36].

### 2.6. Statistical Analysis

A sample size of 45 patients per group was calculated based on an expected mean difference of 0.68 ng/mL and standard deviation (SD) of 1.16 ng/mL in the unOC levels between the fracture and non-fracture groups, providing 80% power at alpha (α) = 0.05 [37]. Participant characteristics, bone outcomes and nutritional parameters were summarised by fracture status using descriptive statistics, and categorical variables as counts (percentages). Normality was assessed within each group using the Shapiro–Wilk test; normally distributed variables are presented as mean ± SD and non-normally distributed variables as median (interquartile range, IQR). As the majority of continuous variables were non-normally distributed in at least one group, between-group comparisons were performed using Mann–Whitney U tests for continuous variables and chi-square tests for categorical variables.

Hierarchical multiple linear regression analyses were conducted to examine the influence of fracture status on bone microarchitecture, strength and turnover markers and to investigate the incremental contribution of nutritional biomarkers [38,39]. Fracture status was coded as fracture = 1 and non-fracture = 2; a positive coefficient therefore indicates a higher outcome value in the non-fracture group. Nutritional biomarkers were examined pathway by pathway in separate hierarchical regression models for each a priori selected bone outcome. In Block 1, fracture status, age, sex, body mass index (BMI), estimated glomerular filtration rate (eGFR) and PTH were entered to account for baseline differences in the bone outcomes. Nutritional biomarkers were tested as (1) vitamin K-dependent carboxylation (Block 2: vitamin K1 and vitamin K2-7; Block 3: cOC, ucOC and unOC); (2) one-carbon metabolism (Block 2: vitamins B6 and B12, and folate; Block 3: homocysteine); (3) vitamin D–calcium homeostasis (Block 2: 25-hydroxyvitamin D and total calcium); (4) antioxidant micronutrients (Block 2: vitamins E and C); (5) protein status (Block 2: albumin); and (6) zinc (Block 2: zinc). 1,25-dihydroxyvitamin D (1,25D) was measured but excluded from the vitamin D–calcium models, as its production is directly regulated by PTH and eGFR, both included in Block 1, precluding independent assessment without collinearity. Block 1 covariates were identical across all models. This pathway-by-pathway approach was adopted to avoid overfitting given the sample size and to preserve the hierarchical structure within each biological pathway. For each hierarchical model, the incremental contribution of each block was quantified by the change in R^2^ (ΔR^2^) with the significance of the F change, reported at the step each block was entered. Unstandardised regression coefficients (β), 95% confidence intervals and *p*-values are reported for all predictors from the final fully adjusted model. The unique variance explained by each individual significant predictor was quantified as the squared semi-partial (part) correlation, reflecting the variance attributable to that predictor independent of all other variables in the model. Model assumptions were evaluated by inspection of residual and normal probability plots, and multicollinearity was assessed using variance inflation factors (VIF); all VIF values ranged from 1.0 to 2.1 (tolerance 0.4–0.8), indicating no evidence of multicollinearity. All analyses were conducted using SPSS (version 29), with statistical significance defined as *p* < 0.05.

Given the biological interrelatedness of bone outcomes obtained from micro-CT and DXA-AHA, established indicators of fracture risk were selected a priori to minimise multicollinearity and reduce the risk of model overfitting. The bone strength parameters included section modulus (indicator of structural strength) [12,35], HAL (indicator of femoral geometry) [40], total hip BMD, femoral neck width, CT and buckling ratio (indicator of cortical bone instability) [6,12]. Trabecular bone microarchitecture parameters included BV/TV, BS/BV (an integrated measure of texture and thickness), Tb.Sp and Tb.Pf (an indicator of surface curvature) [25].

## 3. Results

### 3.1. Patient Characteristics and Unadjusted Between-Group Comparisons

**Figure 1:**
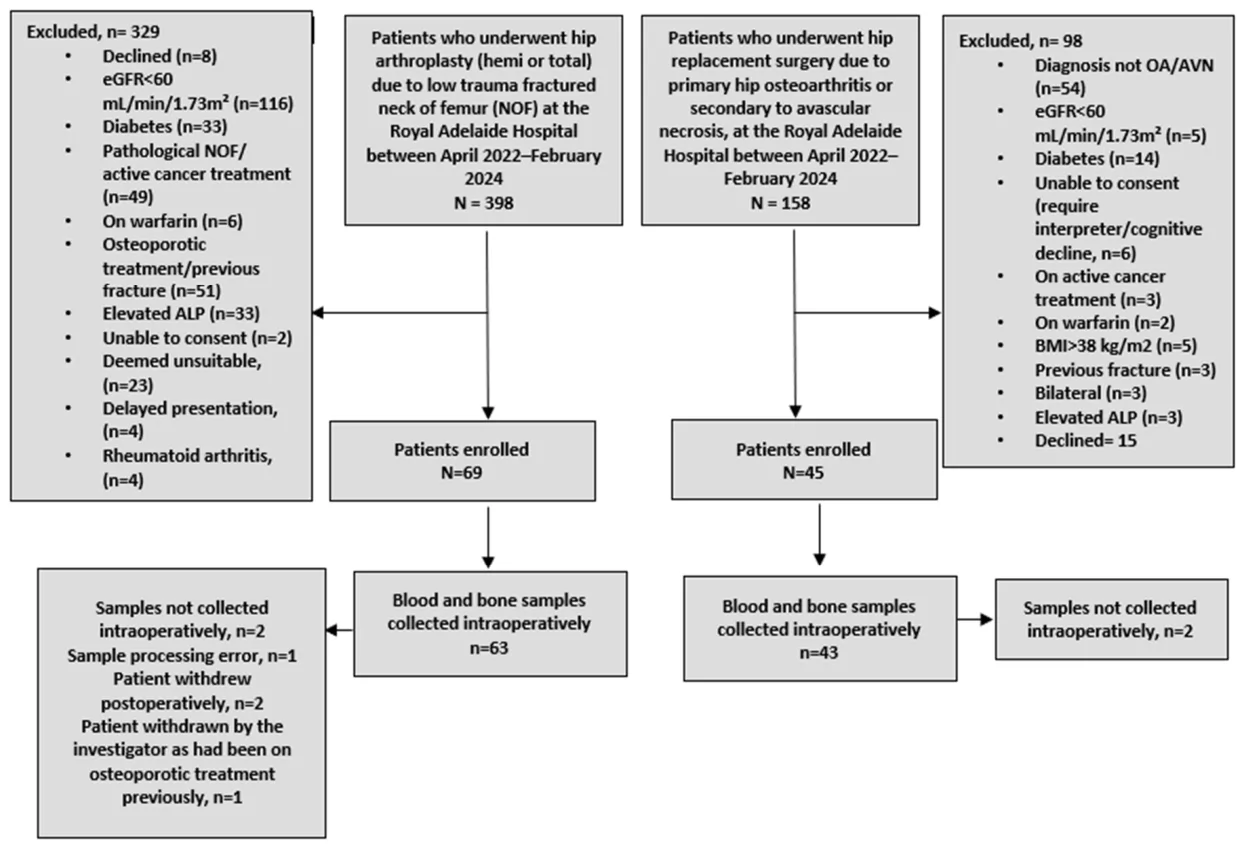
Patient recruitment flowchart.

Fracture group: Three hundred and ninety-eight patients presented for the treatment of hip fracture between April 2022 and February 2024. Of these, 321 patients were not eligible (reasons specified in Figure 1) and 8 declined to participate. Notably, CKD (eGFR < 60 mL/min/1.73 m^2^) was highly prevalent (30%) among the excluded fracture cohort. Sixty-nine patients were enrolled in this study. Of these, blood and bone biopsies were unavailable for six patients (intraoperative specimens could not be collected for two, one sample was processed incorrectly by laboratory staff and two patients withdrew post-operatively). Further, one patient was withdrawn by the investigator due to prior anti-resorptive treatment, which was confirmed through general practitioner records but was not documented in the admission note. Consequently, 63 fracture patients were included in the data analyses. DXA scans could not be performed in twenty-four fracture patients due to postoperative complications.

Non-fracture group: One hundred and fifty-eight patients were scheduled to undergo elective total hip replacement surgery at the Royal Adelaide Hospital between April 2022 and February 2024 for treatment of primary hip osteoarthritis or secondary to avascular necrosis. Ninety-eight patients did not meet the inclusion criteria (Figure 1). Sixty patients were invited to participate, of which 15 declined and 45 (18 females and 27 males) were enrolled in this study as described previously [23]. Intraoperative biopsies could not be collected from two patients, and 43 patients were included in the data analyses. Three patients were unable to undergo DXA scanning due to preoperative unavailability.

Patient characteristics for the fracture and non-fracture groups are presented in Table 2 and Table 3, respectively. The patients in the fracture group were significantly older and had a higher proportion of women compared with the non-fracture group (mean age 81 years vs. 68 years, *p* < 0.001). No clinical cut-offs have been defined for vitamin K1 and vitamin K2-7, but deficiencies of albumin (surrogate for protein status), vitamin D, B12 and vitamin C were prevalent in both groups (Table 2). The remaining nutrients were within the reference range (Table 2). Vitamin D deficiency was similar in the fracture group (35%) and the non-fracture group (36%) whereas deficiencies of albumin (32% vs. 13%), vitamin C (71% vs. 39%) and vitamin B12 (38% vs. 18%) and elevated homocysteine levels (33% vs. 27%) were more prevalent in the fracture compared with the non-fracture group. Physical activity did not differ between groups. Vitamins E and C, cOC and ucOC differed between the groups on unadjusted comparison, with lower cOC and vitamins E and C, and higher ucOC in the fracture group (Table 2). Among BTMs, unadjusted comparisons revealed lower bone ALP (*p* = 0.033) and lower total OC (*p* = 0.003) in the fracture group alongside higher CTX-1 (*p* = 0.026), while PINP did not differ (*p* = 0.54). BMI categories differed significantly between groups (*p* < 0.001). In the fracture group, 4 (6.3%) were underweight (<18.5 kg/m^2^), 40 (63.5%) normal weight, 14 (22.2%) overweight and 5 (7.9%) obese (≥30 kg/m^2^), compared with nil, 11 (25.6%), 12 (27.9%) and 20 (46.5%), respectively, in the non-fracture group.

### 3.2. Adjusted Between-Group Differences in Bone Microarchitecture, Strength and Bone Turnover

After adjustment for age, sex, BMI, eGFR and PTH levels, fracture status was independently associated with hip BMD (β = 0.103, *p* = 0.014), HAL (β = −6.426, *p* = 0.018) and section modulus (β = 261.106, *p* < 0.001) only. After covariate adjustment, fracture status remained independently associated with total OC (β = 5.213, *p* = 0.002) while bone ALP (β = 1.890, *p* = 0.097) showed a trend difference and CTX-1 (*p* = 0.51) was no longer independently associated with fracture status. Addition of ASA grade and physical activity did not influence the results.

### 3.3. Incremental Contributions of Nutritional Biomarker Pathways to Bone Outcomes

In fully adjusted models, no independent associations were observed between bone outcomes and nutritional biomarkers representing one-carbon metabolism, vitamin D–calcium status, antioxidant micronutrients (vitamins E and C), protein or zinc status (*p* > 0.05 for all, Supplementary Table S1), while biomarkers reflecting vitamin K status (Table 4 and Supplementary Table S2) showed significant associations.

Within the vitamin K pathway, the circulating vitamin K block (vitamins K1 and K2-7) did not significantly improve model fit for any bone outcome (ΔR^2^ 0.002–0.044, *p* > 0.05 for all), whereas the OC-fraction block contributed significant incremental variance for five of seven outcomes, indicating that skeletal associations were driven by OC carboxylation status rather than circulating vitamin K concentration (Table 4 and Supplementary Table S2). In fully adjusted models, higher cOC levels were independently and positively associated with section modulus (*p* = 0.011), femoral neck width (*p* = 0.047) and CT shaft (*p* = 0.006) and negatively associated with Tb.Sp (*p* = 0.043) and BS/BV (*p* = 0.049). The variance explained by cOC ranged from 3 to 8%. For Tb.Sp, the osteocalcin-fraction block explained an additional 9.1% of variance (ΔR^2^ = 0.091, *p* = 0.027), within which ucOC was positively associated with Tb.Sp (8.5%, *p* = 0.004) and cOC negatively (4%, *p* = 0.043). For buckling ratio, the OC-fraction block explained a further 11.4% of variance (ΔR^2^ = 0.114, *p* = 0.025), driven by the fully uncarboxylated fraction (unOC; 11.0%, *p* = 0.003). Vitamin K2-7 was negatively associated with HAL, explaining 3.8% of variance (*p* = 0.021). Neither vitamin K1 nor K2-7 was associated with any other bone outcome. For CTX-1, the OC-fraction block explained an additional 34.7% of variance (ΔR^2^ = 0.347, *p* < 0.001); unOC was the dominant predictor (17.3%, *p* < 0.001), with cOC a weaker independent contributor (2.7%, *p* = 0.032).

## 4. Discussion

In this cohort of elderly orthopaedic patients undergoing hip surgery without major metabolic comorbidities, we report three principal findings. First, in metabolically healthy individuals, fracture status was not associated with generalised alterations in bone remodelling activity; rather, fractures are associated with specific deficits in mineralisation and osteocalcin-dependent bone formation pathways, with collagen synthesis markers comparable between groups. Second, among six nutritional pathways examined in fully adjusted models, only vitamin K-dependent biomarkers showed consistent independent associations with bone outcomes, providing insights on the pathway specificity rather than generalised nutritional differences. Third and most mechanistically informative, cOC, ucOC and unOC osteocalcin fractions showed differential associations with distinct aspects of bone quality, a pattern consistent with the functional consequences of impaired vitamin K-dependent carboxylation.

Lower total OC levels and a trend towards lower bone ALP levels in the fracture group after covariate adjustment, alongside comparable CTX-1 and PINP levels between groups, suggest that alterations in fracture patients may be specific to OC-dependent mineralisation pathways rather than generalised bone remodelling alterations. Further, these differences reflect the pre-fracture metabolic state rather than acute fracture-related changes, as bone remodelling markers are reported to rise only 2–6 weeks after fracture [41]. This pattern of OC dysregulation in fracture patients, with lower total OC combined with a higher proportion of inactive fractions (unOC and ucOC) and lower cOC, suggests that the deficit is not simply one of reduced OC production but of impaired carboxylation, resulting in OC that is present but functionally compromised. Consistent with our previous findings in the non-fracture group [23], cOC was positively associated with multiple bone strength outcomes (section modulus, cortical shaft thickness and femoral neck width), in the combined cohort, consistent with its established role in mineral binding and matrix organisation. The partially carboxylated intermediate (ucOC) was associated with increased Tb.Sp, suggesting that partial loss of carboxylation may be sufficient to compromise trabecular microarchitecture. The fully inactive fraction (unOC) was associated positively with cortical bone instability (higher buckling ratio) and bone resorption (CTX-1, explaining approximately 17% of variance), indicating that the fully inactive OC fraction may actively participate in bone loss. These are among the first data to demonstrate outcome-specific associations across OC carboxylation states in a fracture cohort and provide a plausible explanation for higher fracture risk observed in patients with higher unOC and ucOC fractions [37,42]. Direct interrogation of bone compositional properties including mineral crystallinity, collagen crosslinking and mineral-to-matrix organisation using Fourier transform infrared or Raman spectroscopy in specimens stratified by OC carboxylation status would clarify the tissue-level consequences of impaired vitamin K-dependent carboxylation.

Higher vitamin K2-7 was associated with shorter HAL, and higher cOC with greater femoral neck width. The direction of the HAL association is notable, as a large Canadian registry study of over 50,000 women found each standard deviation increase in HAL was associated with 30% higher hip fracture risk (HR: 1.30; 95% CI 1.22–1.38) [40]; shorter HAL in individuals with higher vitamin K2-7 status is therefore in the direction associated with lower fracture risk. Biological plausibility for a relationship between vitamin K2-7 and hip geometry is supported by a randomised trial in postmenopausal women reporting improvements in hip geometry indices, including HAL, with vitamin K2-7 supplementation independent of BMD [43]. However, hip geometry reflects skeletal morphology established over the lifespan, and a single circulating measurement reflects recent rather than cumulative vitamin K status. The present cross-sectional design therefore cannot determine whether vitamin K2-7 influences hip geometry over time, and longitudinal studies with repeated measures would be required to address this question.

Contrary to our secondary hypothesis, one-carbon metabolism and vitamin D–calcium pathways were not independently associated with bone microarchitecture or turnover after full covariate adjustment (Supplementary Table S1). Our findings are in contrast with those of Holstein et al. [44], who reported significantly lower Tb.Th and Tb.N in patients with low serum folate and vitamin B6 concentrations, respectively, compared to those with higher concentrations. It is unclear whether Holstein et al.’s [44] study excluded patients with CKD and/or diabetes mellitus, both of which independently influence bone microarchitecture and strength at the proximal femur [3,5].

No association between other nutritional pathways and bone outcomes in fully adjusted models may reflect a threshold effect. While deficiencies in vitamin C and albumin were prevalent in the fracture group, the limited inter-individual variability across the cohort as a whole may reduce the ability of continuous analyses to identify associations. This does not preclude clinically relevant effects in populations with a higher prevalence of deficiency.

### 4.1. Clinical Implications

These findings carry potential clinical implications for fracture risk assessment and prevention. The independent associations of osteocalcin carboxylation state with bone strength, geometry and microarchitecture, beyond age, sex, BMI, eGFR and PTH, suggest that functional vitamin K biomarkers capture aspects of skeletal fragility not reflected by standard clinical measures. Furthermore, the high prevalence of vitamin C (71%) and albumin (32%) deficiencies observed in the fracture group underscores the nutritional vulnerability of elderly hip fracture patients and highlights the potential value of routine nutritional screening in this population. Collectively, the differential associations of vitamin K-dependent carboxylation status and vitamin K2-7 with bone strength, geometry and microarchitecture are consistent with the inverse associations between vegetable consumption (a dietary source rich in vitamin K) and fracture risk reported in the Rotterdam Study [12] and a GRADE-assessed dose–response meta-analysis [45]. Measurements of cOC, ucOC and unOC using validated immunoassays are not yet widely available in routine clinical settings; however, DXA is widely available and changes in bone strength and geometry can be measured using AHA software. Vitamin K status is modifiable through increased vegetable and fermented food intake or supplementation, and the comprehensive bone outcome endpoints identified in the present study provide a framework for interventional studies examining the effects of vitamin K supplementation on bone health beyond BMD.

### 4.2. Strengths and Limitations

This is the first study reporting circulating vitamin K biomarkers in Australian patients with fragility fracture. The strict exclusion of major secondary causes of fragility fractures including diabetes, CKD, secondary hyperparathyroidism, inflammatory arthritis, cancer or bone-active medications, combined with a comprehensive assessment of bone strength, microarchitecture, turnover and nutritional biomarkers, enabled an integrated evaluation of nutrition–skeletal associations. Hierarchical modelling allowed evaluation of the incremental contribution of six nutritional pathways beyond established demographic determinants, providing insights into pathway-specific contributions to skeletal fragility. Among six physiologically distinct nutritional pathways examined (vitamin K–dependent carboxylation, one-carbon metabolism, vitamin D–calcium homeostasis, antioxidant micronutrients, protein status and zinc), only vitamin K was consistently associated with bone structural outcomes. This pattern of pathway specificity argues against a generalised nutritional confounding explanation and points to vitamin K-dependent carboxylation as a pathway distinctly associated with skeletal fragility in this population. This did not support our secondary hypothesis that one-carbon metabolism and vitamin D–calcium homeostasis would contribute independently, although as discussed above, the restricted range of nutrient concentrations in this cohort limits the inference that can be drawn from these null findings.

However, several limitations warrant consideration. First, the cross-sectional design precludes causal inferences between nutritional factors and fracture risk, and single time-point measurement may not reflect long-term nutritional status, though bone microarchitecture and strength are unlikely to change acutely. The effect of nutrients at the tissue level can only be elucidated using biopsy-based measurements as done in the current investigation, which inherently reflect longer-term skeletal status. Second, although several bone outcomes were measured, the regression analyses focused on a subset to reduce model complexity and avoid overfitting, so potential associations of nutrients with the remaining outcomes were not evaluated. Third, the fracture and non-fracture groups differed substantially in age, sex distribution, residential status and ASA grade, reflecting the inherent clinical differences between these populations. These differences were anticipated and therefore fracture status, age, sex, BMI, eGFR and PTH were included as covariates in all regression models. The addition of ASA grade did not materially alter the regression findings, supporting the robustness of the reported associations. Despite this, residual confounding from unmeasured factors cannot be excluded. In particular, while BMI was included as a covariate, visceral adipose tissue, which more closely reflects adiposity-related bone effects than BMI [46], was not included due to DXA data missing in twenty-four patients in the fracture group. Other unmeasured factors, such as frailty assessment, may also contribute to residual confounding, and the findings should be interpreted as hypothesis-generating rather than confirmatory. Fourth, exclusion of hyperparathyroidism was based on clinical diagnosis documented in medical records rather than biochemical confirmation at enrolment, as intraoperative blood collection was the first opportunity for biochemical assessment. However, PTH levels were measured intraoperatively and included as a covariate in all regression models, partially mitigating this limitation.

## 5. Future Directions

Future studies should incorporate bone material property assessments to provide tissue-level mechanistic insight into OC fraction roles in skeletal fragility. Given the high prevalence of CKD among the excluded fracture cohort (30%), examination of vitamin K-dependent mechanisms and nutrient-based prevention strategies in this high-risk population warrants urgent investigation.

## 6. Conclusions

In conclusion, in the absence of major metabolic disease, skeletal fragility is characterised by specific deficits in vitamin K-dependent mineralisation pathways rather than generalised bone remodelling dysregulation. Among six nutritional pathways examined, only vitamin K-dependent osteocalcin carboxylation consistently predicted bone outcomes, with carboxylated, undercarboxylated and fully uncarboxylated fractions showing differential associations with bone strength, geometry and microarchitecture that reflect a biological gradient from active-matrix regulation to resorption. These findings identify vitamin K-dependent carboxylation as a mechanistically specific, clinically informative and potentially modifiable factor associated with skeletal fragility, warranting targeted investigation of vitamin K optimisation as a fracture prevention strategy.

## Figures and Tables

**Table 1:** Exclusion criteria.

Patients were excluded if they were/had: Unable to provide written informed consent and caregiver unavailable eGFR < 60 mL/min/1.73 m^2^ Elevated alkaline phosphatase (>110 U/L) Diabetes mellitus Primary or secondary hyperparathyroidism Inflammatory arthritis Cancer (current/past three years) Previous fragility fracture Antiresorptive treatment Warfarin therapy Medications affecting bone metabolism BMI > 38 kg/m^2^

**Table 2:** Patient characteristics: fracture and non-fracture groups.

Parameter (mean ± SD) or Median (IQR)	Fracture Group (*n* = 63)	Non-Fracture Group (*n* = 43)	*p*-Values (Unadjusted)	Reference Values
Age	81 ± 9	68 ± 9	<0.001	-
Sex, no (%)			<0.001	-
Females	48 (76%)	18 (42%)		
Males	15 (24%)	25 (58%)		
Residential status, *n* (%) (prior to surgery)			<0.001	-
Private/independent	45 (71)	43 (100)		*-*
Nursing home	16 (25)	0		
Retirement village	1 (2)	0		
Respite care	1 (2)	0		
Body mass index (kg/m^2^)	24.2 ± 3.9	29.3 ± 6.4	<0.001	25
eGFR, mL/min/1.73 m^2^	79 (68, 86)	89 (79, 90)	<0.001	>60
ASA grade			<0.001	
Normal	1	3		
Mild systemic disease	12	24		
Severe systemic disease	42	14		
Severe systemic disease constant threat to life	8	0		
Total calcium (mmol/L)	2.30 ± 0.12	2.37 ± 0.10	0.002	2.10–2.60
Ionised calcium (mmol/L)	1.20 (1.17, 1.23)	1.21 (1.18, 1.23)	0.67	1.1–1.3
Albumin (g/L)	35 (33, 38)	38 (36, 40)	<0.001	34–48
Vitamin D, 25-hydroxyvitamin D (nmol/L)	59.4 ± 26.6	52.6 ± 21.6	0.215	60–160
Alkaline phosphatase (U/L) ^#^	85 (74, 102)	87 (66, 101)	0.522	30–110
1,25D (pmol/L)	95 (67, 133)	103 (80.4, 123)	0.534	48–190
Vitamin B1 (nmol/L)	149.5 (117, 176)	143 (126.5, 164)	0.947	70–200
Vitamin B2 (nmol/L)	240 ± 35	246 ± 41	0.531	174–471
Vitamin B6 (nmol/L)	78 (63, 108)	73 (64.5, 91.2)	0.452	35–110
Vitamin B12 (pmol/L)	309 (216, 411)	308 (247, 401)	0.662	≥260
Folic acid (nmol/L)	25 (15, 36)	25.8 (18, 35.3)	0.673	6–45
Homocysteine (µmol/L)	12 (8, 16)	12 (10, 15)	0.732	4–14
Vitamin E (µmol/L)	27 (20, 33)	29 (23, 40)	0.046	12–46
Vitamin C (µmol/L)	14 (8.5, 24)	26 (8.5, 48.75)	0.026	23–114
Zinc (µmol/L)	9.9 (6.2, 14.9)	10.4 (7.2, 15.7)	0.297	9–21
Vitamin K1 (nmol/L)	0.42 (0.27, 0.74)	0.52 (0.35, 1.02)	0.113	0.35–2.5
Vitamin K2-7 (nmol/L)	0.20 (0.13, 0.34)	0.25 (0.19, 0.34)	0.179	Not available
cOC (ng/mL)	5.19 ± 2.4	6.92 ± 2.68	<0.001	Not available
unOC (ng/mL)	4.02 ± 3.15	4.18 ± 2.66	0.419	Not available
ucOC (ng/mL)	6.46 (4.05, 8.31)	7.43 (5.76, 9.57)	0.015	Not available
Magnesium (mmol/L)	0.79 ± 0.10	0.78 ± 0.14	0.803	0.7–1.10
Total lymphocytes (×10^9^/L)	1.12 (0.87, 1.66)	1.56 (1.09, 2.05)	0.009	1.10–3.5

Abbreviations: eGFR, estimated glomerular filtration rate; 1,25D, 1,25-dihydroxyvitamin D; cOC, carboxylated osteocalcin; unOC, uncarboxylated osteocalcin; ucOC, undercarboxylated osteocalcin. ^#^ ALP values reflect intraoperative measurements. Patients were screened using pre-admission values (<110 U/L). Intraoperative values that exceeded this threshold normalised within 1–2 days post-operatively, consistent with acute physiological response to surgical stress.

**Table 3:** Bone parameters: fracture and non-fracture groups.

Parameter (mean ± SD)	Fracture Group (*n* = 63)	Non-Fracture Group (*n* = 43)	*p*-Values (Unadjusted)	Reference Values
Trabecular bone microarchitecture				Not available
BV/TV (%)	7.55 (6.22, 9.72)	9.37 (7.74, 11.7)	0.004	
BS/BV (mm^−1^)	24.36 ± 2.91	23.36 ± 3.46	0.125	
Tb.Th (mm)	0.15 (0.14, 0.17)	0.15 (0.13, 0.17)	0.976	
Tb.Sp (mm^−1^)	1.11 (0.93, 1.20)	1.06 (0.94, 1.16)	0.275	
Tb.N (mm^−1^)	0.51 (0.41, 0.64)	0.64 (0.51, 0.72)	0.003	
Tb.Pf (mm^−1^)	7.03 ± 1.36	5.96 ± 1.68	<0.001	
SMI	1.73 ± 0.25	1.52 ± 0.30	<0.001	
Connectivity density (mm^−3^)	2.10 (1.72, 3.1)	2.74 (2.18, 3.29)	0.009	
Unplate index	1.82 ± 0.10	1.77 ± 0.10	0.007	
DXA derived bone strength				
Total hip BMD (g/cm^2^)	0.79 ± 0.12	1.0 ± 0.18	<0.001	
Hip axis length	113.1 (108.6, 121.0)	116.4 (105.5, 124.9)	0.503	113.45 ± 5.92
Femoral neck width (mm)	37.68 ± 4.70	40.67 ± 7.00	0.04	32.96 ± 2.23
CT, neck (mm)	3.98 (3.22, 5.58)	5.79 (4.44, 6.59)	0.001	Females: 5.23 ± 1.85 [6] Males: 5.47 ± 2.28 [6]
CT, calcar (mm)	3.57 ± 1.32	4.97 ± 2.29	0.004	Females: 5.09 ± 1.30 [6] Males: 5.83 ± 1.82 [6]
CT, shaft (mm)	4.50 (3.90, 5.70)	5.41 (4.05, 6.32)	0.033	Females: 7.44 ± 2.07 [6] Males: 7.74 ± 2.88 [6]
CSA (mm^2^)	122 ± 25	193.5 ± 61.5	<0.001	157.58 ± 22.49
Section modulus (mm^3^)	506 (451, 689)	973.3 (687.5, 1168.0)	<0.001	740.65 ± 140.89
CSMI (mm^4^)	8969 (7951, 14,194)	19642 (11274, 25540)	<0.001	13,666.65 ± 3141.58
Strength index	1.39 (1.11, 1.56)	1.71 (1.38, 2.29)	<0.001	1.58 ± 0.42
Buckling ratio	4.69 (3.24, 5.47)	3.87 (3.07, 4.74)	0.102	Females: 3.63 ± 1.42 [6] Males: 4.26 ± 1.92 [6]
Bone remodelling markers				
Bone ALP (µg/L)	10.8 (8.1, 13.6)	12.1 (9.5, 16.3)	0.033	Not available
PINP (µg/L)	36 (28, 51)	37 (27, 72)	0.54	15–80 ug/L
CTX-1 (ng/L)	548 (380, 713)	433 (294, 573)	0.026	0–399 ng/L
Total osteocalcin (ng/mL)	15.4 (11.3, 19.4)	19.6 (14.8, 21.6)	0.003	Not available
Parathyroid hormone (pmol/L)	4 (3.2, 5.4)	4.5 (3.7, 5.8)	0.16	0.8–5.5

Abbreviations: BV/TV, bone volume to tissue volume; BS/BV, bone surface to bone volume; Tb.Th, trabecular thickness; Tb.Sp, trabecular separation; Tb.N, trabecular number; Tb.Pf, trabecular pattern factor; SMI, Structure Model Index; CSA, cross-sectional area, CSMI, cross-sectional moment of inertia; CT, cortical thickness; CTX-1, *C*-terminal cross-linking telopeptide of type I collagen; PINP, procollagen type I *N*-propeptide; Bone ALP, bone alkaline phosphatase.

**Table 4:** Hierarchical regression results: vitamin K-dependent carboxylation.

Bone Outcome	Adjusted R^2^	Significant Predictor Unstandardised β (95% CI); *p*-Value
Buckling ratio	0.121	unOC: 0.238 (0.085, 0.391); *p* = 0.003
Tb.Sp	0.057	ucOC: 0.057 (0.019, 0.095); *p* = 0.004 cOC: −0.049 (−0.096, −0.002); *p* = 0.043
BS/BV	0.075	cOC: −0.328 (−0.655, −0.001); *p* = 0.049
Section Modulus	0.588	cOC: 33.27 (7.858, 58.696); *p* = 0.011
Femoral neck width	0.444	cOC: 0.509 (0.007, 1.011); *p* = 0.047
CT (shaft)	0.214	cOC: 0.252 (0.074, 0.429); *p* = 0.006
HAL	0.483	Vitamin K2-7: −10.86 (−20.00, −1.72); *p* = 0.021
CTX-1	0.434	unOC: 41.396 (26.608, 56.184); *p* < 0.001

Abbreviations: cOC, carboxylated osteocalcin; unOC, uncarboxylated osteocalcin; ucOC, undercarboxylated osteocalcin; Tb.Sp, trabecular separation; BS/BV, bone surface/bone volume; CT, cortical thickness; HAL, hip axis length; CTX-1, *C*-terminal telopeptide of type I collagen. Adjusted R^2^ (Model 3; adjusted for age, sex, BMI, fracture status, PTH, eGFR).

## Data Availability

Data described in this manuscript will be made available in a de-identified manner upon request to the corresponding author and approval from the Central Adelaide Local Health Network (CALHN) ethics committee.

## Supplementary Materials

The following supporting information can be downloaded at https://www.mdpi.com/article/10.3390/nu18162663/s1, Table S1: Vitamin D and One-carbon Metabolism: Hierarchical Regression Results; Table S2: Hierarchical regression results for Vitamin K Pathway.

## Abbreviations

The following abbreviations are used in this manuscript:

OC	Osteocalcin
cOC	Carboxylated osteocalcin
ucOC	Undercarboxylated osteocalcin
unOC	Uncarboxylated osteocalcin
CTX-1	*C*-terminal cross-linking telopeptide of type I collagen
PINP	Procollagen type I *N*-propeptide
Bone ALP	Bone alkaline phosphatase
