# Supplementary material for: Vitamin K Status and Skeletal Fragility: Differential Associations of Osteocalcin Carboxylation States with Bone Strength, Geometry and Microarchitecture
Source: Nutrients. 2026 Aug 14;18(16):2663. doi: 10.3390/nu18162663 (PMC13516068; doi:10.3390/nu18162663)
Supplement: Supplementary file 1 [file nutrients-18-02663-s001.zip › nutrients-4401380-supplementary.pdf]

Table S1: Vitamin D and One-carbon Metabolism: Hierarchical Regression Results

Outcome	Predictor	Unstandardised β	95% CI	p-value	R^2	ΔR^2	p-value (Sig F)
**Vitamin D-Calcium Pathway**							
**Bone volume/tissue volume (BV/TV), (n =106)**							
Block 1: Demographic and clinical covariates							
	Fracture status	0.899	-0.633, 2.430	0.247	0.146	0.146	0.018
	Age (years)	0.017	-0.053, 0.088	0.623			
	Sex	1.539	0.319, 2.760	0.014			
	BMI (kg/m^2)	0.040	-0.070, 0.151	0.469			
	eGFR (ml/mom/1.73m^2)	0.009	-0.059, 0.078	0.785			
	PTH (pmol/L)	0.038	-0.243, 0.318	0.791			
Block 2: Vitamin D-Calcium					0.156	0.010	0.576
	Vitamin D (nmol/L)	-0.010	-0.034, 0.013	0.378			
	Total calcium (mmol/L)	1.722	-3.551, 6.994	0.518			
**Bone surface/bone volume (BS/BV)**							
Block 1: Demographic and clinical covariates					0.092	0.092	0.152
	Fracture status	0.257	-1.462, 1.976	0.768			
	Age (years)	0.026	-0.053, 0.105	0.520			
	Sex	-0.942	-2.313, 0.429	0.176			
	BMI (kg/m^2)	-0.046	-0.171, 078	0.459			
	eGFR (ml/mom/1.73m^2)	-0.023	-0.100, 0.054	0.557			
	PTH (pmol/L)	0.250	-0.064, 0.565	0.118			
Block 2: Vitamin D-Calcium					0.106	0.014	0.487
	Vitamin D (nmol/L)	0.006	-0.020, 0.032	0.661			
	Total calcium (mmol/L)	-3.450	-9.370, 2.470	0.250			
**Trabecular separation (n =106)**							
Block 1: Demographic and clinical covariates							
	Fracture status	-0.048	-0.303, 0.207	0.708	0.044	0.044	0.634
	Age (years)	-0.002	-0.014, 0.009	0.691			
	Sex	-0.128	-0.332, 0.075	0.213			
	BMI (kg/m^2)	0.003	-0.015, 0.022	0.734			
	eGFR (ml/mom/1.73m^2)	0.001	-0.011, 0.012	0.885			
	PTH (pmol/L)	-0.036	-0.082, 0.011	0.132			
					0.048	0.004	0.806

Block 2: Vitamin D-Calcium	Vitamin D (nmol/L)	0.00	-0.004, 0.003	0.803			
	Total calcium (mmol/L)	-0.257	-1.135, 0.620	0.562			
**Trabecular pattern factor (n =106)**							
Block 1: Demographic and clinical covariates					0.201	0.201	0.001
	Fracture status	-0.522	-1.332, 0.288	0.204			
	Age (years)	2.166e-6	-0.037, 0.037	1.0			
	Sex	0.974	-1.620, -0.328	0.004			
	BMI (kg/m^2)	-0.017	-0.076, 0.041	0.562			
	eGFR (ml/mom/1.73m^2)	-0.012	-0.048, 0.024	0.512			
	PTH (pmol/L)	0.014	-0.134, 0.162	0.852			
Block 2: Vitamin D-Calcium					0.206	0.005	0.750
	Vitamin D (nmol/L)	0.004	-0.008, 0.016	0.534			
	Total calcium (mmol/L)	-0.688	-3.477, 2.101	0.626			
**Total Hip Bone Mineral Density (n =79)**							
Block 1: Demographic and clinical covariates					0.533	0.533	<0.001
	Fracture status	0.104	0.019, 0.188	0.017			
	Age (years)	-0.003	-0.007, 0.002	0.203			
	Sex	0.137	0.068, 0.206	<0.001			
	BMI (kg/m^2)	0.007	0.002, 0.013	0.013			
	eGFR (ml/mom/1.73m^2)	0.001	-0.003, 0.005	0.767			
	PTH (pmol/L)	-0.009	-0.024, 0.005	0.209			
					0.534	0.001	0.902
Block 2: Vitamin D-Calcium	Vitamin D (nmol/L)	0.000	-0.001, 0.002	0.663			
	Total calcium (mmol/L)	-0.024	-0.302, 0.255	0.866			
**Buckling ratio (n= 79)**							
Block 1: Demographic and clinical covariates					0.114	0.114	0.176
	Fracture status	-0.770	-1.840, 0.299	0.155			
	Age (years)	0.026	-0.027, 0.079	0.331			
	Sex	0.821	-0.039, 1.682	0.061			
	BMI (kg/m^2)	-0.043	-0.115, 0.029	0.240			
	eGFR (ml/mom/1.73m^2)	0.009	-0.041, 0.059	0.722			
	PTH (pmol/L)	-0.029	-0.219, 0.160	0.757			
Block 2: Vitamin D-Calcium					0.134	0.020	0.444
	Vitamin D (nmol/L)	-0.001	-0.018, 0.015	0.881			

	Total calcium (mmol/L)	2.255	-1.253, 5.763	0.204			
**Section Modulus (n=79)**							
Block 1: Demographic and clinical covariates					0.594	0.594	<0.001
	Fracture status	269.286	119.603, 418.968	<0.001			
	Age (years)	0.281	-7.157, 7.719	0.940			
	Sex	368.242	247.784, 488.701	<0.001			
	BMI (kg/m^2)	4.859	-5.225, 14.942	0.340			
	eGFR (ml/mom/1.73m^2)	-0.120	-7.166, 6.925	0.973			
	PTH (pmol/L)	4.254	-22.274, 30.783	0.750			
Block 2: Vitamin D-Calcium					0.603	0.009	0.468
	Vitamin D (nmol/L)	-1.281	-3.587, 1.025	0.272			
	Total calcium (mmol/L)	-102.790	-593.705, 388.126	0.678			
**Femoral Neck Width (n =79)**							
					0.437	0.437	<0.001
Block 1: Demographic and clinical covariates	Fracture status	0.179	-2.852, 3.211	0.906			
	Age (years)	0.070	-0.081, 0.220	0.360			
	Sex	7.730	5.291, 10.170	<0.001			
	BMI (kg/m^2)	0.130	-0.074, 0.334	0.209			
	eGFR (mL/min/1.73m^2)	0.008	-0.134, 0.151	0.908			
	PTH (pmol/L)	0.234	-0.303, 0.771	0.388			
					0.438	0.002	0.909
Block 2: Vitamin D-Calcium	Vitamin D (nmol/L)	-0.009	-0.055, 0.038	0.709			
	Total calcium (mmol/L)	1.344	-8.598, 11.285	0.788			
**Cortical Thickness (shaft), (n =79)**							
					0.186	0.186	0.019
Block 1: Demographic and clinical covariates	Fracture status	0.270	-0.801, 1.342	0.617			
	Age (years)	0.022	-0.032, 0.075	0.421			
	Sex	1.278	0.416, 2.141	0.004			
	BMI (kg/m^2)	0.047	-0.025, 0.119	0.200			
	eGFR (mL/min/1.73m^2)	0.017	-0.034, 0.067	0.505			
	PTH (pmol/L)	0.050	-0.140, 0.240	0.601			
					0.197	0.011	0.623
Block 2: Vitamin D-Calcium	Vitamin D (nmol/L)	0.006	-0.011, 0.022	0.495			

	Total calcium (mmol/L)	1.071	-2.444, 4.585	0.546			
**Hip Axis Length (n = 79)**							
Block 1: Demographic and clinical covariates					0.492	0.492	<0.001
	Fracture status	-5.706	-11.074, -0.338	0.038			
	Age (years)	-0.024	-0.290, 0.243	0.859			
	Sex	16.199	11.896, 20.502	<0.001			
	BMI (kg/m²)	0.364	0.003, 0.725	0.048			
	eGFR (mL/min/1.73m²)	-0.075	-0.332, 0.181	0.561			
	PTH (pmol/L)	-0.273	-1.260, 0.714	0.583			
Block 2: Vitamin D-Calcium	Vitamin D (nmol/L)	-0.028	-0.112, 0.055	0.499	0.508	0.017	0.317
	Total calcium (mmol/L)	-11.295	-28.842, 6.251	0.203			
**CTX-1 (n = 106)**							
					0.118	0.118	0.054
Block 1: Demographic and clinical covariates	Fracture status	-71.078	-205.489, 63.333	0.469			
	Age (years)	-4.074	-10.263, 2.115	0.296			
	Sex	-48.753	-155.930, 58.423	0.194			
	BMI (kg/m²)	-9.611	-19.345, 0.123	0.369			
	eGFR (mL/min/1.73m²)	-6.464	-12.5, 0.428	0.053			
	PTH (pmol/L)	2.000	-22.64, 26.64	0.036			
				0.872	0.172	0.054	0.048
Block 2: Vitamin D-Calcium	Vitamin D (nmol/L)	-1.072	-3.1, 0.956	0.297			
	Total calcium (mmol/L)	544.102	89.489, 998.715	0.020			
**P1NP (n = 106)**					0.028	0.028	0.843
Block 1: Demographic and clinical covariates	Fracture status	8.165	-17.11, 33.44	0.523			
	Age (years)	0.133	-1.026, 1.293	0.820			
	Sex	-0.313	-20.735, 20.108	0.976			
	BMI (kg/m²)	-0.476	-2.298, 1.346	0.605			
	eGFR (mL/min/1.73m²)	0.039	-1.092, 1.170	0.946			
	PTH (pmol/L)	1.310	-3.310, 5.930	0.575			
					0.099	0.072	0.028
Block 2: Vitamin D-Calcium	Vitamin D (nmol/L)	-0.413	-0.791, -0.035	0.033			

	Total calcium (mmol/L)	81.390	-4.106, 166.885	0.062			
**Bone ALP (n = 106)**							
Block 1: Demographic and clinical covariates					0.105	0.105	0.092
	Fracture status	1.702	-0.550, 3.954	0.137			
	Age (years)	0.015	-0.089,0.118	0.777			
	Sex	0.181	-1.611, 1.974	0.841			
	BMI (kg/m^2)	-0.006	-0.169, 0.157	0.939			
	eGFR (ml/mom/1.73m^2)	0.042	-0.060, 0.144	0.420			
	PTH (pmol/L)	0.286	-0.139, 0.711	0.184			
Block 2: Vitamin D-Calcium					0.135	0.030	0.207
	Vitamin D (nmol/L)	-0.028	-0.062, 0.006	0.108			
	Total calcium (mmol/L)	3.575	-4.034, 11.184	0.353			
**One Carbon Metabolism**							
**Bone volume/tissue volume (BV/TV) (n =106)**							
Block 1: Demographic and clinical covariates					0.150	0.150	0.0161
	Fracture status	0.910	-0.979, 2.798	0.340			
	Age (years)	0.009	-0.074, 0.091	0.838			
	Sex	1.525	0.071, 2.979	0.040			
	BMI (kg/m^2)	0.068	-0.069, 0.205	0.328			
	eGFR (ml/mom/1.73m^2)	0.000	-0.085, 0.085	0.996			
	PTH (pmol/L)	-0.066	-0.407, 0.275	0.701			
Block 2: B-vitamin status					0.151	0.001	0.994
	Vitamin B6 (nmol/L)	-0.002	-0.019, 0.015	0.795			
	Folate(nmol/L)	0.005	-0.062, 0.072	0.885			
	Vitamin B12 (pmol/L)	-4.52e-5	-0.003, 0.003	0.977			
Block 3: Homocysteine	Homocysteine (µmol/L)	0.006	-0.108, 0.121	0.911	0.151	0.000	0.911
**Bone surface/bone volume (BS/BV) (n =106)**							
Block 1: Demographic and clinical covariates					0.118	0.118	0.160
	Fracture status	0.172	-1.936, 2.280	0.871			
	Age (years)	0.031	-0.062, 0.123	0.510			
	Sex	-0.896	-2.520, 0.727	0.274			
	BMI (kg/m^2)	-0.058	-0.211, 0.096	0.456			
	eGFR (ml/mom/1.73m^2)	-0.026	-0.121, 0.069	0.587			
	PTH (pmol/L)	0.299	-0.082, 0.680	0.122			
					0.120	0.002	0.984

Block 2: B-vitamin status	Vitamin B6(nmol/L)	-0.001	-0.020, 0.018	0.952			
	Folate (nmol/L)	0.025	-0.050, 0.100	0.510			
	Vitamin B12 (pmol/L)	0.001	-0.003, 0.004	0.758			
Block 3: Homocysteine					0.145	0.025	0.161
	Homocysteine (μmol/L)	0.091	-0.037, 0.219	0.161			
**Trabecular separation (n =106)**							
Block 1: Demographic and clinical covariates					0.051	0.051	0.699
	Fracture status	-0.082	-0.415, 0.252	0.627			
	Age (years)	0.000	-0.015, 0.014	0.946			
	Sex	-0.163	-0.420, 0.094	0.211			
	BMI (kg/m^2)	0.001	-0.023, 0.025	0.928			
	eGFR ($ml/mom/1.73m^2$)	0.004	-0.011, 0.019	0.636			
	PTH (pmol/L)	-0.037	-0.097, 0.023	0.227			
Block 2: B-vitamins status					0.076	0.025	0.603
	Vitamin B6(nmol/L)	-0.001	-0.004, 0.002	0.502			
	Folate(nmol/L)	-0.002	-0.014, 0.010	0.765			
	Vitamin B12(pmol/L)	0.000	-0.001, 0.000	0.277			
Block3: Homocysteine	Homocysteine (μmol/L)	-0.008	-0.028, 0.012	0.425	0.084	0.009	0.425
**Trabecular pattern factor (n =106)**							
Block 1: Demographic and clinical covariates					0.180	0.180	0.023
	Fracture status	-0.322	-1.323, 0.680	0.524			
	Age (years)	0.003	-0.041, 0.047	0.884			
	Sex	-0.925	-1.696, -0.154	0.019			
	BMI (kg/m^2)	-0.019	-0.092, 0.053	0.597			
	eGFR ($ml/mom/1.73m^2$)	-0.025	-0.070, 0.020	0.271			
	PTH (pmol/L)	0.052	-0.129, 0.233	0.566			
Block 2: B-vitamins status					0.191	0.012	0.802
	Vitamin B6(nmol/L)	4.893e-6	-0.009, 0.009	0.999			
	Folate(nmol/L)	-0.001	-0.036, 0.035	0.969			
	Vitamin B12 (pmol/L)	0.001	-0.001, 0.002	0.324			
Block3: Homocysteine							
	Homocysteine (μmol/L)	0.005	-0.056, 0.066	0.878	0.192	0.000	0.878
**Total hip BMD (n= 79)**							
Block 1: Demographic and clinical covariates					0.583	0.583	<0.001
	Fracture status	0.046	-0.039, 0.132	0.284			
	Age (years)	-0.001	-0.005, 0.003	0.658			
	Sex	0.124	0.059, 0.189	<0.001			
	BMI (kg/m^2)	0.013	0.007, 0.019	<0.001			

	eGFR (ml/mom/1.73m ^{2})	0.002	-0.002, 0.006	0.320			
	PTH (pmol/L)	-0.013	-0.027, 0.001	0.074			
					0.595	0.012	0.648
Block 2: B-vitamins status	Vitamin B6(nmol/L)	0.000	-0.001, 0.000	0.476			
	Folate(nmol/L)	0.001	-0.003, 0.004	0.627			
	Vitamin B12 (pmol/L)	0.000	0.000, 0.000	0.207			
Block3: Homocysteine	Homocysteine (μmol/L)	0.004	-0.002, 0.010	0.176	0.608	0.013	0.176
**Buckling ratio (n= 79)**							
Block 1: Demographic and clinical covariates					0.118	0.118	0.247
	Fracture status	-0.819	-2.068, 0.431	0.195			
	Age (years)	0.018	-0.037, 0.072	0.515			
	Sex	0.900	-0.036, 1.836	0.059			
	BMI (kg/m ^{2})	-0.009	-0.093, 0.074	0.821			
	eGFR (ml/mom/1.73m ^{2})	0.005	-0.051, 0.062	0.852			
	PTH (pmol/L)	-0.100	-0.309, 0.108	0.340			
Block 2: B-vitamins status					0.140	0.023	0.673
	Vitamin B6(nmol/L)	-0.002	-0.012, 0.009	0.721			
	Folate(nmol/L)	0.023	-0.024, 0.071	0.333			
	Vitamin B12 (pmol/L)	0.001	-0.002, 0.004	0.434			
Block3: Homocysteine	Homocysteine(μ mol/L)	0.035	-0.051, 0.121	0.419	0.150	0.010	0.419
**Section Modulus (n=79)**							
Block 1: Demographic and clinical covariates					0.660	0.660	<0.001
	Fracture status	212.536	68.054, 357.018	0.005			
	Age (years)	3.218	-3.077, 9.512	0.310			
	Sex	358.016	249.773, 466.259	<0.001			
	BMI (kg/m ^{2})	10.684	1.037, 20.331	0.031			
	eGFR (ml/mom/1.73m ^{2})	1.557	-4.987, 8.101	0.636			
	PTH (pmol/L)	6.100	-18.030, 30.230	0.615			
Block 2: B-vitamins status					0.670	0.010	0.646
	Vitamin B6(nmol/L)	0.486	-0.722, 1.693	0.424			
	Folate(nmol/L)	-1.854	-7.345, 3.638	0.502			
	Vitamin B12 (pmol/L)	0.018	-0.291, 0.327	0.908			
Block 3: Homocysteine	Homocysteine (μmol/L)	4.844	-5.110, 14.797	0.334	0.675	0.005	0.334
**Femoral Neck Width (n =79)**							

Block 1: Demographic and clinical covariates					0.509	0.509	<0.001
	Fracture status	0.198	-2.906, 3.301	0.899			
	Age (years)	0.074	-0.061, 0.209	0.278			
	Sex	7.740	5.415, 10.065	<0.001			
	BMI (kg/m²)	0.174	-0.033, 0.382	0.097			
	eGFR (mL/min/1.73m²)	-0.038	-0.178, 0.103	0.594			
	PTH (pmol/L)	0.163	-0.355, 0.681	0.532			
Block 2: B-vitamins status					0.547	0.037	0.199
	Vitamin B6(nmol/L)	0.027	0.001, 0.053	0.042			
	Folate (nmol/L)	-0.006	-0.124, 0.112	0.914			
	Vitamin B12	-0.001	-0.008, 0.005	0.708			
Block 3: Homocysteine	Homocysteine (μmol/L)	0.084	-0.130, 0.297	0.437	0.552	0.005	0.437
**Cortical Thickness (n=79)**							
Block 1: Demographic and clinical covariates					0.175	0.175	0.059
	Fracture status	0.648	-0.615, 1.911	0.309			
	Age (years)	0.029	-0.027, 0.084	0.304			
	Sex	0.982	0.036, 1.929	0.042			
	BMI (kg/m²)	0.054	-0.030, 0.138	0.205			
	eGFR (mL/min/1.73m²)	0.016	-0.041, 0.073	0.579			
	PTH (pmol/L)	0.061	-0.150, 0.272	0.565			
					0.273	0.097	0.061
Block 2: B-vitamins status	Vitamin B6(nmol/L)	0.007	-0.004, 0.017	0.213			
	Folate(nmol/L)	-0.050	-0.098, -0.002	0.040			
	Vitamin B12 (pmol/L)	0.002	-0.001, 0.004	0.204			
Block 3: Homocysteine	Homocysteine (μmol/L)	0.026	-0.061, 0.113	0.547	0.277	0.005	0.547
**Hip Axis Length (n = 79)**							
					0.491	0.491	<0.001
Block 1: Demographic and clinical covariates	Fracture status	-6.161	-12.523, 0.202	0.057			
	Age (years)	0.052	-0.226, 0.329	0.711			
	Sex	15.952	11.185, 20.718	<0.001			
	BMI (kg/m²)	0.320	-0.105, 0.745	0.137			
	eGFR (mL/min/1.73m²)	0.007	-0.281, 0.296	0.959			
	PTH (pmol/L)	-0.088	-1.150, 0.975	0.869			
Block 2: B-vitamins status					0.504	0.013	0.673
	Vitamin B6(nmol/L)	0.023	-0.030, 0.077	0.382			
	Folate(nmol/L)	-0.112	-0.354, 0.130	0.358			

	Vitamin B12 (pmol/L)	-0.004	-0.0170, 0.010	0.604			
Block 3: Homocysteine	Homocysteine (µmol/L)	-0.122	-0.561, 0.316	0.578	0.507	0.003	0.578
**CTX-1 (n = 106)**							
					0.085	0.085	0.355
Block 1: Demographic and clinical covariates	Fracture status	-9.209	-166.171, 147.752	0.907			
	Age (years)	-3.416	-10.306, 3.473	0.326			
	Sex	-70.099	-190.929, 50.730	0.251			
	BMI (kg/m²)	-10.248	-21.668, 1.173	0.078			
	eGFR (mL/min/1.73m²)	-4.610	-11.636, 2.415	0.195			
	PTH (pmol/L)	7.044	-21.358, 35.446	0.622			
Block 2: B-vitamins status	Vitamin B6(nmol/L)	0.406	-1.015, 1.827	0.570	0.112	0.027	0.542
	Folate(nmol/L)	-0.605	-6.156, 4.945	0.828			
	Vitamin B12 (pmol/L)	-0.186	-0.446, 0.073	0.156			
Block 3: Homocysteine	Homocysteine (µmol/L)	-3.857	-13.421, 5.706	0.424	0.121	0.008	0.424
**P1NP (n = 106)**							
Block 1: Demographic and clinical covariates					0.021	0.021	0.954
	Fracture status	13.056	-20.087, 46.2	0.435			
	Age (years)	0.056	-1.399, 1.510	0.939			
	Sex	-2.534	-28.048, 22.981	0.844			
	BMI (kg/m²)	-0.695	-3.107, 1.717	0.567			
	eGFR (mL/min/1.73m²)	0.359	-1.125, 1.842	0.631			
	PTH (pmol/L)	0.204	-5.794, 6.201	0.946			
Block 2: B-vitamins status	Vitamin B6(nmol/L)	0.021	-0.279, 0.321	0.887	0.069	0.048	0.313
	Folate(nmol/L)	-0.020	-1.192, 1.152	0.973			
	Vitamin B12 (pmol/L)	-0.049	-0.104, 0.005	0.076			
Block 3: Homocysteine	Homocysteine (µmol/L)	0.956	-1.064, 2.975	0.348	0.081	0.012	0.348
**Bone ALP (n = 106)**							
Block 1: Demographic and clinical covariates					0.141	0.141	0.076
	Fracture status	2.813	0.149, 5.477	0.039			
	Age (years)	0.043	-0.074, 0.160	0.467			
	Sex	0.456	-1.595, 2.506	0.659			
	BMI (kg/m²)	-0.072	-0.266, 0.122	0.461			
	eGFR (mL/min/1.73m²)	0.064	-0.055, 0.184	0.285			
	PTH (pmol/L)	0.342	-0.140, 0.824	0.161			
					0.165	0.024	0.581
Block 2: B-vitamins status	Vitamin B6(nmol/L)	-0.002	-0.026, 0.023	0.900			

	Folate(nmol/L)	-0.018	-0.112, 0.076	0.705			
	Vitamin B12 (pmol/L)	-0.002	-0.007, 0.002	0.279			
Block 3: Homocysteine	Homocysteine (μmol/L)	0.077	-0.086, 0.239	0.349	0.176	0.011	0.349

Unstandardised regression coefficients (β), 95% confidence intervals and p-values are reported from the final fully adjusted model for all predictors (Model 2 for the vitamin D pathway and Model 3 for the one-carbon metabolism). R^2, ΔR^2 and significance of F change reflect the incremental contribution of each block at the step it was entered.: cOC, carboxylated, osteocalcin; unOC, uncarboxylated osteocalcin; ucOC, undercarboxylated osteocalcin; CTX-1, C-terminal telopeptide of type I collagen, PINP, procollagen type I N-propeptide; Bone ALP, bone alkaline phosphatase.

Table S2: Hierarchical regression results for Vitamin K Pathway

Outcome	Predictor	Unstandardised β	95% CI	p-value	R^2	ΔR^2	p-value (Sig F)
**Bone volume/tissue volume (BV/TV), (n =106)**							
Block 1: Demographic and clinical covariates					0.178	0.178	0.005
	Fracture status	0.665	-0.870, 2.20	0.392			
	Age (years)	-0.006	-0.072, 0.060	0.857			
	Sex	1.562	0.386, 2.738	0.010			
	BMI (kg/m^2)	0.061	-0.042, 0.165	0.243			
	eGFR ($ml/mom/1.73m^2$)	.000	-0.0670. 067	0.994			
	PTH (pmol/L)	-0.093	-0.372, 0.186	0.510			
Block 2: Vitamin K status					0.208	0.030	0.188
	Vitamin K1 (nmol/L)	0.637	-0.195, 1.470	0.132			
	Vitamin K2-7 (nmol/L)	-1.593	-4.115, 0.930	0.213			
Block 3: OC fractions					0.248	0.040	0.205
	cOC	0.283	0.007, 0.558	0.044			
	ucOC	-0.081	-0.302, 0.140	0.687			
	unOC	-0.041	-0.241, 0.159	0.470			
**Bone surface/bone volume (BS/BV), (n =106)**							
Block 1: Demographic and clinical covariates					0.098	0.098	0.133
	Fracture status	0.946	-0.877, 2.770	0.305			
	Age (years)	0.052	-0.026, 0.130	0.191			
	Sex	-0.661	-2.058, 0.736	0.350			
	BMI (kg/m^2)	-0.069	-0.192, 0.055	0.272			
	eGFR ($ml/mom/1.73m^2$)	-0.036	-0.116, 0.044	0.375			
	PTH (pmol/L)	0.358	0.027, 0.690	0.034			
Block 2: Vitamin K status					0.100	0.002	0.907
	Vitamin K1	-0.045	-1.034, 0.944	0.928			
	Vitamin k2	1.196	-1.801, 4.193	0.430			
Block 3: OC fractions					0.177	0.077	0.047
	cOC	-0.328	-0.655, -0.001	0.049			
	ucOC	-0.062	-0.325, 0.201	0.641			
	unOC	0.034	-0.204, 0.272	0.778			
**Trabecular separation (Tb.Sp) (n =106)**							
Block 1: Demographic and clinical covariates					0.039	0.039	0.706
	Fracture status	-0.105	-0.367,0.158	0.4			
	Age (years)	-0.003	-0.014, 0.009	0.6			
	Sex	-0.157	-0.358, 0.044	0.1			
	BMI (kg/m^2)	-0.001	-0.019, 0.017	0.8			

	eGFR (ml/mom/1.73m^2)	0.005	-0.006, 0.017	0.4			
	PTH (pmol/L)	-0.038	-0.086, 0.009	0.112			
Block 2: Vitamin K status					0.070	0.031	0.224
	Vitamin K1	-0.087	-0.229, 0.056	0.229			
	Vitamin k2	0.237	-0.194, 0.668	0.278			
Block 3: OC fractions					0.161	0.091	0.027
	cOC	-0.049	-0.096, -0.002	0.043			
	ucOC	0.057	0.019, 0.095	0.004			
	unOC	0.006	-0.028, 0.040	0.734			
**Trabecular pattern factor, (Tb.Pf) (n =106)**							
Block 1: Demographic and clinical covariates					0.204	0.204	0.001
	Fracture status	-0.291	-1.158, 0.576	0.507			
	Age (years)	0.006	-0.031, 0.043	0.751			
	Sex	-0.964	-1.628, -0.299	0.005			
	BMI (kg/m^2)	-0.026	-0.084, 0.033	0.384			
	eGFR (ml/mom/1.73m^2)	-0.018	-0.056, 0.020	0.357			
	PTH (pmol/L)	0.055	-0.103, 0.212	0.492			
Block 2: Vitamin K status					0.209	0.005	0.770
	Vitamin K1	-0.208	-0.678, 0.262	0.382			
	Vitamin K2	-0.362	-1.062, 1.787	0.615			
Block 3: OC fractions					0.249	0.041	0.199
	cOC	-0.046	-0.202, 0.109	0.557			
	ucOC	-0.052	-0.176, 0.073	0.414			
	unOC	-0.072	-0.185, 0.041	0.210			
**Total hip Bone Mineral Density (Total Hip BMD), (n= 79)**							
Block 1: Demographic and clinical covariates					0.529	0.529	<0.001
	Fracture status	0.093	0.010, 0.175	0.028			
	Age (years)	-0.003	-0.007, 0.001	0.106			
	Sex	0.161	0.095,0.227	<0.001			
	BMI (kg/m^2)	0.008	0.003, 0.013	0..004			
	eGFR (ml/mom/1.73m^2)	-0.001	-0.005, 0.003	0.621			
	PTH (pmol/L)	-0.008	-0.023, 0.006	0.261			
					0.582	0.053	0.018
Block 2: Vitamin K status	Vitamin K1	0.057	0.013, 0.100	0.012			
	Vitamin k2	-0.021	-0.159, 0.118	0.764			
	cOC	0.012	-0.002, 0.025	0.101	0.619	0.037	0.116

Block 3: OC fractions	ucOC	-0.014	-0.026, -0.002	0.022			
	unOC	-0.007	-0.019, 0.006	0.280			
**Buckling ratio (n= 79)**							
Block 1: Demographic and clinical covariates					0.124	0.124	0.140
	Fracture status	-0.647	-1.695, 0.400	0.221			
	Age (years)	0.025	-0.024, 0.074	0.308			
	Sex	0.834	0.007, 1.660	0.048			
	BMI (kg/m^2)	-0.019	-0.087, 0.049	0.580			
	eGFR (ml/mom/1.73m^2)	-0.003	-0.051, 0.046	0.916			
	PTH (pmol/L)	-0.081	-0.271, 0.108	0.393			
Block 2: Vitamin K status					0.133	0.009	0.692
	Vitamin K1	-0.089	-0.635, 0.457	0.746			
	Vitamin K2	0.048	-1.707, 1.804	0.956			
Block 3: OC fractions	cOC	-0.037	-0.212, 0.138	0.678	0.247	0.114	0.025
	ucOC	0.036	-0.113, 0.185	0.634			
	unOC	0.238	0.085, 0.391	0.003			
**Section Modulus (n=79)**							
Block 1: Demographic and clinical covariates					0.591	0.591	<0.001
	Fracture status	201.398	49.262, 353.534	0.010			
	Age (years)	0.425	-6.634, 7.484	0.905			
	Sex	391.26	271.168, 511.363	<0.001			
	BMI (kg/m^2)	6.821	-3.076, 16.718	0.173			
	eGFR (ml/mom/1.73m^2)	0.853	-6.229, 7.935	0.811			
	PTH (pmol/L)	6.111	-21.370, 33.592	0.659			
Block 2: Vitamin K status					0.593	0.002	0.871
	Vitamin K1	0.948	-78.369, 80.265	0.981			
	Vitamin K2	41.898	-213.217, 297.014	0.744			
Block 3: OC fractions					0.647	0.055	0.023
	cOC	33.277	7.858, 58.696	0.011			
	ucOC	-10.671	-32.340, 10.998	0.329			
	unOC	9.738	-12.558, 32.034	0.386			
**Femoral Neck Width (n =79)**							
Block 1: Demographic and clinical covariates	Fracture status	-0.401	-3.406, 2.604	0.791	0.456	0.456	<0.001
	Age (years)	0.057	-.083, 0.196	0.418			
	Sex	7.636	5.264, 10.008	<0.001			
	BMI (kg/m^2)	0.180	-.015, 0.376	0.070			
	eGFR (mL/min/1.73m^2)	0.006	-0.134, 0.146	0.935			
	PTH (pmol/L)	0.095	-0.448, 0.638	0.728			
	Vitamin K1 (nmol/L)	-0.949	-2.516, 0.618	0.231	0.468	0.012	0.463

Block 2: Vitamin K status	Vitamin K2-7 (nmol/L)	0.673	-4.367, 5.712	0.791			
Block 3: OC fractions	cOC (ng/mL)	0.509	.007, 1.011	0.047	0.524	0.056	0.062
	ucOC (ng/mL)	-0.055	-.483, 0.373	0.798			
	unOC (ng/mL)	0.197	-.244, 0.637	0.375			
**Cortical Thickness (n=79)**							
					0.187	0.187	0.019
Block 1: Demographic and clinical covariates	Fracture status	-0.223	-1.285, 0.840	0.677			
	Age (years)	0.008	-0.041, 0.057	0.744			
	Sex	1.324	0.486, 2.163	0.002			
	BMI (kg/m²)	0.062	-0.007, 0.131	0.077			
	eGFR (mL/min/1.73m²)	0.005	-0.044, 0.055	0.826			
	PTH (pmol/L)	-0.012	-0.204, 0.180	0.902			
Block 2: Vitamin K status	Vitamin K1 (nmol/L)	0.376	-0.178, 0.930	0.180	0.231	0.044	0.146
	Vitamin K2-7 (nmol/L)	0.533	-1.249, 2.314	0.553			
Block 3: OC fractions	cOC (ng/mL)	0.252	0.074, 0.429	0.006	0.327	0.095	0.032
	ucOC (ng/mL)	-0.051	-0.203, 0.100	0.501			
	unOC (ng/mL)	-0.046	-0.201, 0.110	0.561			
**Hip Axis Length, (HAL), (n = 79)**							
					0.492	0.492	<0.001
Block 1: Demographic and clinical covariates	Fracture status	-6.981	-12.432, -1.529	0.013			
	Age (years)	-0.013	-0.266, 0.239	0.916			
	Sex	15.128	10.824, 19.431	<0.001			
	BMI (kg/m²)	0.276	-0.078, 0.631	0.124			
	eGFR (mL/min/1.73m²)	0.114	-0.140, 0.367	0.375			
	PTH (pmol/L)	-0.25	-1.235, 0.735	0.614			
Block 2: Vitamin K status	Vitamin K1 (nmol/L)	-0.124	-2.967, 2.718	0.931	0.520	0.028	0.139
	Vitamin K2-7 (nmol/L)	-10.865	-20.007, -1.723	0.021			
Block 3: OC fractions	cOC (ng/mL)	0.162	-0.749, 1.073	0.723	0.557	0.037	0.149
	ucOC (ng/mL)	-0.425	-1.224, 0.374	0.292			

	unOC (ng/mL)	0.591	-0.186, 1.367	0.133			
**CTX-1 (n = 106)**							
					0.134	0.134	0.030
Block 1: Demographic and clinical covariates	Fracture status	-139.288	-252.296, -26.279	0.016			
	Age (years)	-5.353	-10.252, -0.454	0.033			
	Sex	-76.524	-163.662, 10.614	0.084			
	BMI (kg/m²)	-4.338	-12.026, 3.350	0.265			
	eGFR (mL/min/1.73m²)	-3.858	-8.824, 1.107	0.126			
	PTH (pmol/L)	-2.104	-22.771, 18.563	0.840			
					0.148	0.014	0.458
Block 2: Vitamin K status	Vitamin K1 (nmol/L)	-23.422	-85.326, 38.482	0.454			
	Vitamin K2-7 (nmol/L)	-42.867	-227.731, 141.997	0.646			
					0.495	0.347	<0.001
Block 3: OC fractions	cOC (ng/mL)	22.152	1.929, 42.376	0.032			
	ucOC (ng/mL)	12.686	-3.556, 28.928	0.124			
	unOC (ng/mL)	41.396	26.608, 56.184	<0.001			
**P1NP (n = 106)**					0.031	0.031	0.809
Block 1: Demographic and clinical covariates	Fracture status	8.423	-18.67, 35.51	0.538			
	Age (years)	-0.032	-1.208, 1.145	0.958			
	Sex	-3.356	-24.723, 18.011	0.756			
	BMI (kg/m²)	0.081	-1.756, 1.917	0.931			
	eGFR (mL/min/1.73m²)	0.308	-0.883, 1.500	0.608			
	PTH (pmol/L)	2.045	-2.905, 6.994	0.414			
Block 2: Vitamin K status	Vitamin K1 (nmol/L)	-7.503	-22.639, 7.633	0.327	0.051	0.020	0.390
	Vitamin K2-7 (nmol/L)	-13.589	-57.791, 30.613	0.543			
Block 3: OC fractions	cOC (ng/mL)	2.287	-2.567, 7.141	0.352	0.131	0.080	0.050
	ucOC (ng/mL)	-0.416	-4.305, 3.473	0.832			
	unOC (ng/mL)	3.770	0.138, 7.403	0.042			
**Bone ALP, (n = 106)**							
Block 1: Demographic and clinical covariates					0.103	0.103	0.103
	Fracture status	0.835	-1.414, 3.085	0.463			
	Age (years)	0.005	-0.092, 0.103	0.911			
	Sex	0.047	-1.688, 1.782	0.957			

	BMI (kg/m²)	0.029	-0.124, 0.182	0.709			
	eGFR (mL/min/1.73m²)	0.078	-0.021, 0.177	0.120			
	PTH (pmol/L)	0.231	-0.180, 0.642	0.268	0.150	0.047	0.081
Block 2: Vitamin K status	Vitamin K1 (nmol/L)	-1.222	-2.454, 0.010	0.052			
	Vitamin K2-7 (nmol/L)	1.445	-2.235, 5.125	0.437			
Block 3: OC fractions	cOC (ng/mL)	0.301	-0.102, 0.703	0.141	0.266	0.115	0.004
	ucOC (ng/mL)	0.181	-0.142, 0.504	0.269			
	unOC (ng/mL)	0.274	-0.021, 0.568	0.068			

Unstandardised regression coefficients (β), 95% confidence intervals and p-values are reported from the final fully adjusted model (Model 3) for all predictors. R^2, ΔR^2 and significance of F change reflect the incremental contribution of each block at the step it was entered.
Abbreviations: cOC, carboxylated osteocalcin; unOC, uncarboxylated osteocalcin; ucOC, undercarboxylated osteocalcin; Tb.Sp, trabecular separation; CT, cortical thickness; HAL, hip axis length; CTX-1, C-terminal telopeptide of type I collagen; PINP, procollagen type I N-propeptide; Bone ALP, bone alkaline phosphatase.
